# A Surgical Novelty for Preventing Gossypiboma

**DOI:** 10.7759/cureus.93020

**Published:** 2025-09-23

**Authors:** Rajesh Rajendran, Karthikeyan Srinivasan, Niyas Ahamed

**Affiliations:** 1 Urology, KAP Viswanatham Medical College, Trichy, IND; 2 Surgical Gastroenterology, Madurai Medical College, Madurai, IND; 3 Surgical Gastroenterology, Kalaignar Centenary Super Specialty Hospital, Guindy, Chennai, IND

**Keywords:** gossypiboma, novelty, sponge count, stress free postop, tied surgical sponge

## Abstract

Background

Gossypiboma, the term used for a retained surgical sponge during surgery, is deemed to be an act of negligence. Though considered to be a rare event, the exact occurrence goes under the carpet due to fear of embarrassment, loss of reputation to the treating personnel, and also the medicolegal consequences. Verifying the surgical sponges used during surgery, although it may appear straightforward, might be a daunting task, especially during prolonged and complicated surgeries. A simpler and effective technique to handle surgical sponges has been put forward in this study aimed at reducing the risk of gossypiboma and ensuring intraoperative safety.

Methodology

A multicentric study was conducted from three different hospitals over a period of three years, from 2021 to 2024. A total of 398 patients who underwent laparotomy surgeries of both emergency and elective types were enrolled in the study. A novel technique of handling the surgical sponge was put into use. The ease and practicality of using such a modified technique was surveyed among the surgical crew members (n=33) post-operatively using a structured questionnaire.

Results and conclusion

No case of gossypiboma was reported during the study. The surgical crew members have found this technique easy and handy to implement, thereby reducing their physical and mental strain. This is evident from the survey results that had a highly significant p-value emphasising the usefulness of this technique. While several newer techniques have been proposed to prevent gossypiboma, this new cost-effective and hassle-free alternative offers to enhance surgical safety without adding to the patient's financial burden.

## Introduction

Gossypiboma, also known as textiloma, refers to a mass of cotton matrix that has been retained within the body during surgery. The term *gossypium* is derived from Latin, meaning "cotton", and *boma* in Swahili means "place of concealment" [1]. Surgical sponges account for 70% of gossypiboma cases, making it the most common form of retained foreign body [2]. While the intra-abdominal cavity is the most frequent site, gossypibomas have also been reported in the cranial cavity, thoracic cavity, and breast [3-7].

The clinical presentation and complications of gossypiboma are highly variable. As a foreign body, the pathological process starts off with a granulomatous reaction, initiating an inflammatory cascade that may ultimately lead to abscess formation [8]. Any delay in diagnosis and treatment can result in significant morbidity and, in some cases, even mortality.

As per the literature, the incidence of this occurrence ranges from one in 1000 to one in 32672 surgeries [8]. However, the true incidence of gossypiboma may not be apparent due to underreporting. Decreased reporting of such a complication is likely due to the fear of medicolegal implications, professional embarrassment and humiliation to the surgical team [9]. All these consequences can damage the hospital's reputation, potentially leading to legal proceedings under the doctrine of res ipsa loquitur, and resulting in compensation being awarded to the affected patient.

Retained surgical sponge within the abdominal cavity, though an uncommon surgical error, is still a preventable occurrence [1,10]. Identifying a blood-soaked sponge during surgery can be a strenuous task, more so during emergency surgery, as it is often difficult to distinguish between a blood-soaked sponge from actual or clotted blood per se [2]. To avoid this turmoil, several technical innovations have been introduced to make sponge count an easy job for the surgical crew.

In this study, we have instituted a more efficient and conducive technique for handling and counting surgical sponges during surgery, which has been aimed at reducing the risk of retained surgical sponges.

Aim and objectives

The aims were to assess the feasibility and acceptance of a novel sponge-handling technique among surgical crew members in order to minimise the risk of retained surgical sponge.

## Materials and methods

This was a multicenter, prospective observational study that was conducted over a period of three years, between May 1st 2021 to April 30th 2024, after obtaining ethical committee clearance from the Institutional Ethical Committee at Madurai Medical College (approval 23012023). Three institutions participated in the study, namely: Aiyshwariya Hospital in Trichy, Department of Urology at KAPV Medical College in Trichy, and Sri Gowri Health Care Centre in Madurai. A total of 398 patients (n=398) were enrolled. Laparotomy cases performed in both elective and emergency settings across the three centers were taken into account. Patients who did not consent were excluded from the study.

Procedure

**Figure 1:**
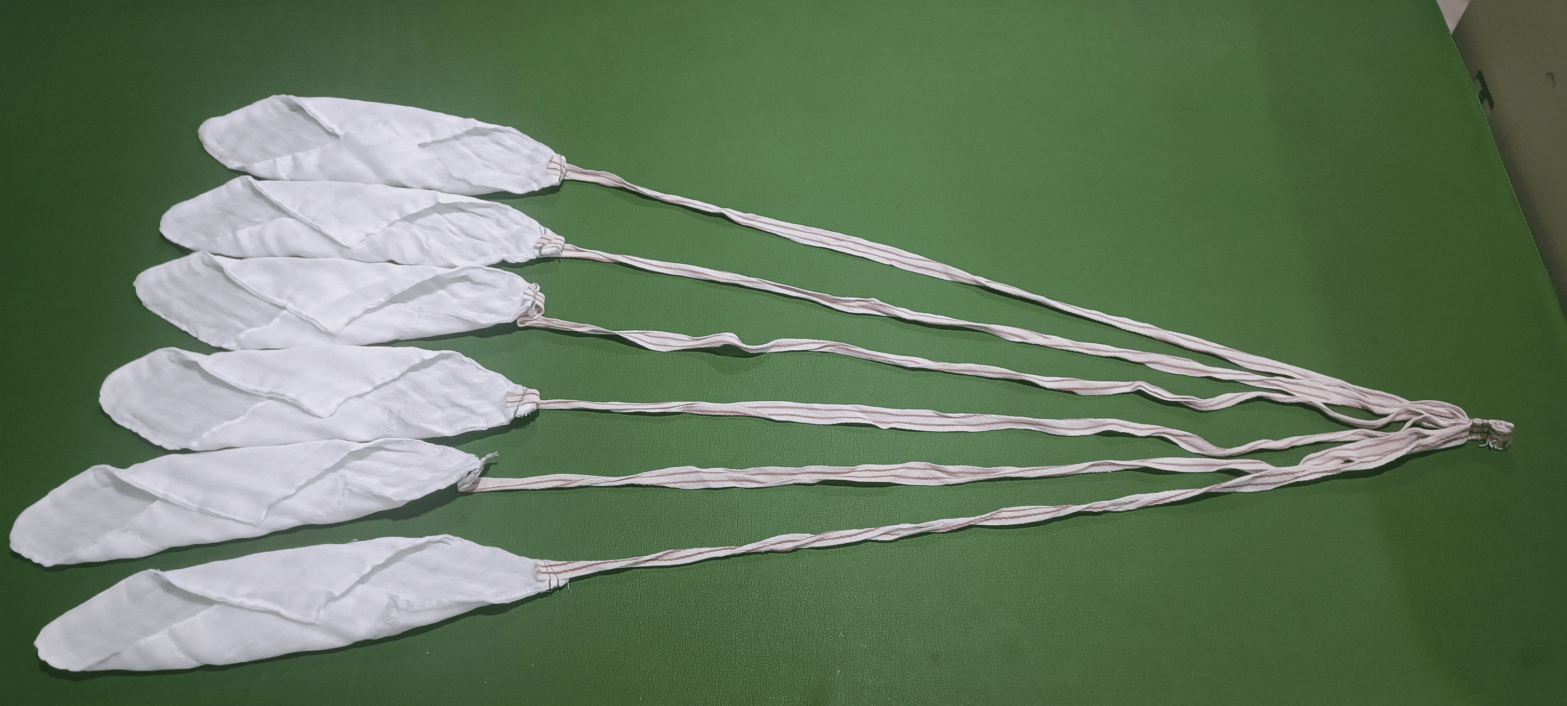
Newer method of using surgical sponges (all five sponges tied together at their tail end)

**Figure 2:**
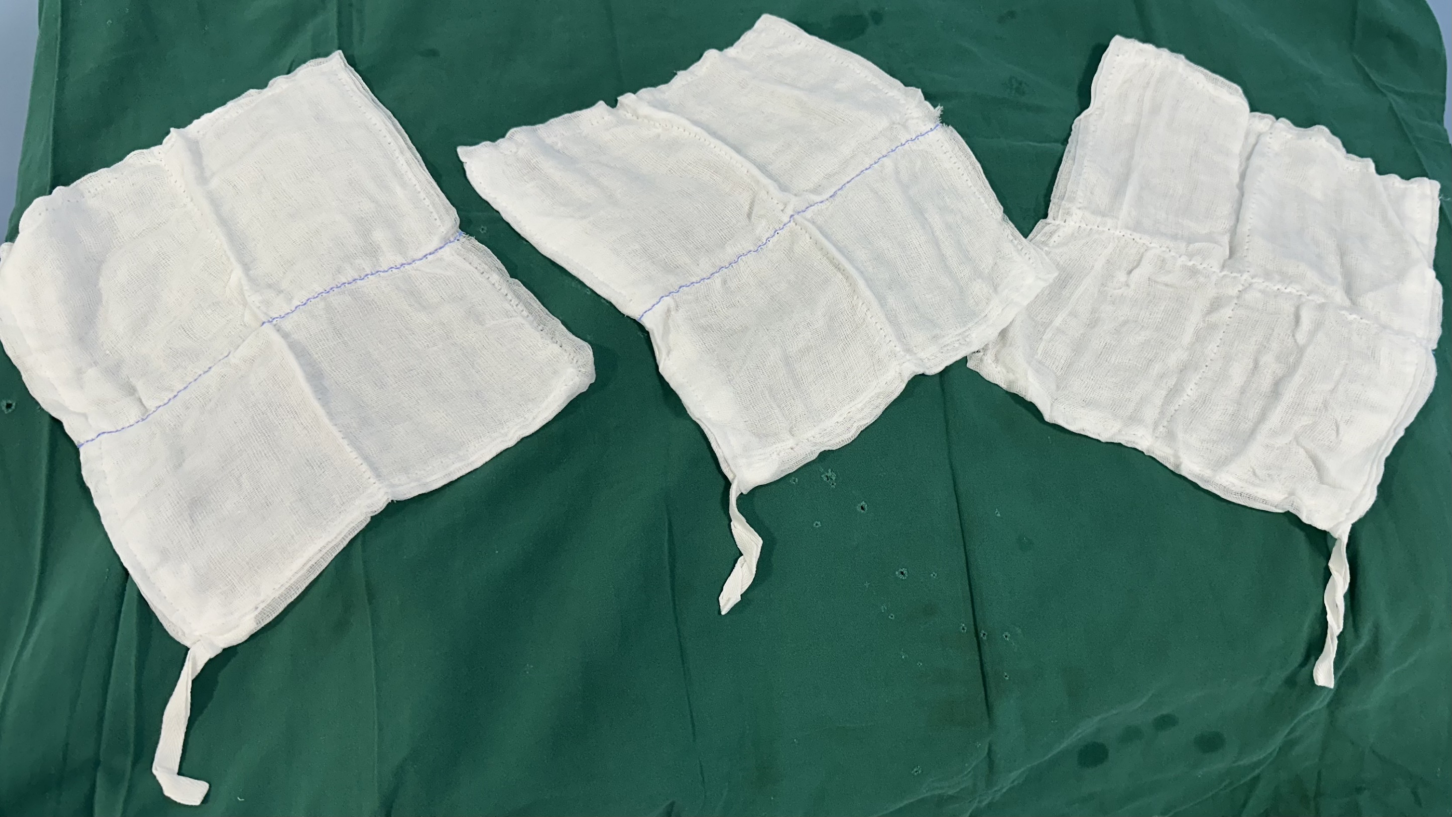
Older (conventional) method of handling surgical sponges

In this study, the conventional use of surgical sponges (length of tail end of conventional sponge is 10cm) was modified. Instead of using single sponges, we utilized packs of five sponges that were knotted together at their tail ends (length of the tail end of sponge here is 40cm) (Figures 1, 2). During laparotomy, as soon as the peritoneum was opened, one pack (containing five sponges tied together at their tail end) was introduced to absorb blood and peritoneal secretions. Once the pack of five sponges had been drenched with blood or peritoneal secretions, the entire pack was replaced by a fresh set of five sponges. This technique did enable the surgical crew, in particular, the scrub nurse and the surgeon, to maintain better control and accountability of the sponge count throughout the procedure. To make sure none of them were retained, sponge count was performed towards the end of the surgery.

Following surgery, to assess the feasibility and acceptance of this novel sponge-handling technique, all surgical team members (n=33) were surveyed on their experience of using this technique. A survey questionnaire was designed to compare the usage of the conventional sponge count technique with the newer technique that was implemented in this study. The scoring for each question ranges from 0 to 10 (Appendix 1). The data obtained was analyzed with IBM SPSS software version 29.0 (IBM Corp., Armonk, NY, USA). Descriptive statistics, the mean, median, Interquartile Range (IQR) and Standard Deviation (SD), were used to describe continuous variables. The normality of the data was verified with Shapiro-Wilk's test. To find the significant difference between the bivariate samples in paired groups, the Wilcoxon Signed-rank test was used and represented with a Whiskers-Box Plot. In the above statistical tool, the probability value of <0.05 is considered statistically significant.

## Results

In this study a total of 398 patients were enrolled, of which 242 (60.8%) were male and 156 (39.2%) were female. The institution-wise breakup was 212 patients (53.3%) from the Department of Urology in KAP Viswanatham Medical College, Trichy; 123 patients (30.9%) from Aiyshwariya Hospital, Trichy; and 63 patients (15.8%) from Sri Gowri Health Care Centre, Madurai (Table 1).

**Table 1:** Place of surgery

S.No	Institution	Patients (n)
1	Aiyshwariya Hospital, Trichy	212 (53.3%)
2	KAP Viswanatham Medical College, Trichy	123 (30.9%)
3	Sri Gowri Health Care Centre, Madurai	63 (15.8%)

The surgical procedures that were performed include hysterectomy, prostatectomy, radical cystectomy, nephrectomy, genitourinary trauma surgery, upper gastrointestinal tract surgery, hepato-pancreatico-biliary surgery, and lower gastrointestinal surgery (Table 2). Of the 398 cases, 301 patients (75.6%) underwent elective surgeries, while 97 patients (24.4%) underwent emergency surgical procedures.

**Table 2:** Type of surgery performed HPB – hepato-pancreatico-biliary; GI – gastrointestinal

S.No	Type of Surgery	Patients (n)
1	Prostatectomy	83 (20.9%)
2	Hysterectomy	71 (17.8%)
3	HPB surgery	66 (16.6%)
4	Upper GI Surgery	49 (12.3%)
5	Nephrectomy	44 (11%)
6	Radical Cystectomy	38 (9.5%)
7	Genitourinary Trauma Surgery	27 (6.7%)
8	Lower GI Surgery	20 (5%)

No case of gossypiboma was reported during the study period or in their subsequent follow-up. A total of 33 surgical team members (20 nurses and 13 surgeons) completed the end-of-study survey which compared both, the new and older methods of handling the surgical sponge. Their overall experience was assessed with the survey questionnaire and the results were analyzed thereafter (Table 3).

**Table 3:** Survey results Old – Old method; New – New method; Q1 – Questionnaire 1; Q2 – Questionnaire 2; Std – Standard; IQR – Interquartile Range

Statistics					
		Old Q1	Old Q2	New Q1	New Q2
N	Valid	33	33	33	33
Mean		8.1	9.3	9.4	9.5
Median		8.0	9.0	9.0	9.0
Std. Deviation		0.7	0.5	0.5	0.5
Range		3.0	2.0	1.0	1.0
IQR		0.0	1.0	1.0	1.0
Percentiles	25	8.0	9.0	9.0	9.0
	50	8.0	9.0	9.0	9.0
	75	8.0	10.0	10.0	10.0

**Figure 3:**
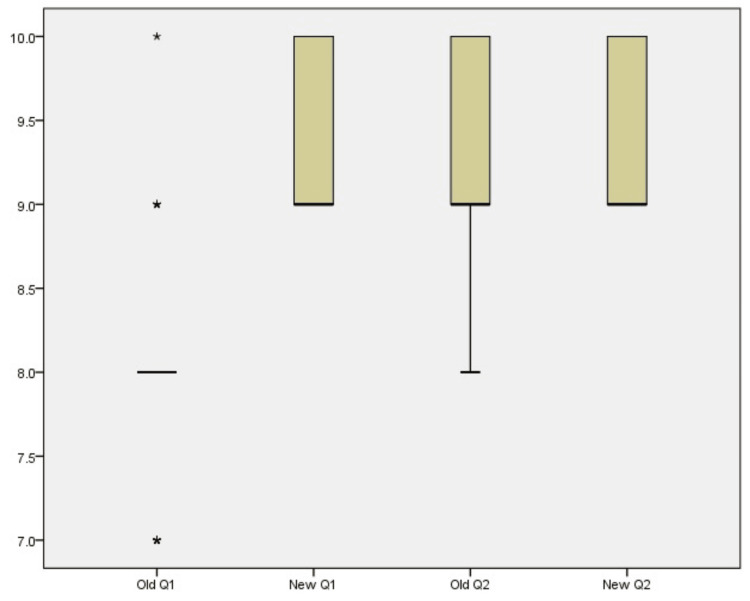
Whiskers-Box plot between old and new method Old – Old method; New – New method; Q1 – Questionnaire 1; Q2 – Questionnaire 2

The significant difference between the bivariate samples has been represented with the Whiskers-Box Plot (Figure 3). The ease of sponge counting in the perioperative period was significantly higher with the new method, as evident from a p-value of 0.0005. Moreover, the confidence among team members in ensuring that no sponge was retained was notably higher with the new technique (p = 0.034) (Table 4).

**Table 4:** Statistical significance (p-value) ^a^ Wilcoxon Signed Ranks Test; ^b^ Based on negative ranks Old – Old method; New – New method; Q1 – Questionnaire 1; Q2 – Questionnaire 2

Test Statistics^a^		
Q	Z	p-value
New Q1 - Old Q1	-4.539^b^	0.0005
New Q2 - Old Q2	-2.121^b^	0.034

The team members felt that the newer technique was more useful, especially in cases with intense blood loss where the sponge count can cause mayhem.

## Discussion

A retained surgical sponge during surgery, although rare, is an unacceptable and certainly avoidable error. Gossypiboma can result in significant postoperative complications, as well as legal proceedings for the caregivers. The rarity of the issue might be owed to the underreporting of the incidence by the hospital/surgical fraternity due to its medicolegal implications and aftermath. Hence, the true incidence remains uncertain.

The likelihood of gossypiboma increases during emergency surgery, when the operated patient is obese, when there is a deviation from the planned procedure, during prolonged surgeries, involvement of multiple surgical crew members and with the failure to record the exact surgical sponge count [11,12].

There are four stages through which gossypiboma progresses. To begin with, it is the foreign body reaction that occurs, followed by secondary infection, mass formation and remodelling. Foreign body reaction may vary from a fibrinous response to an exudative process that can lead to abscess formation [13]. The small bowel remains the most common site of adhesion and foreign body reaction owing to its thin wall and large surface area. Although 30% of patients remain asymptomatic, the clinical presentation depends on the anatomical location and the pathological process involved [14].

Preventive measures against gossypiboma include staff education on strictly conforming to the WHO theatre checklist [15], tagging of radio-opaque markers to the surgical sponges, and perioperative counts of sponges multiple times to ensure the avoidance of any retained foreign material.

Newer modalities have been described to decrease the incidence of gossypiboma. To name a few, adding a radiofrequency tag to the surgical items used and scanning the surgical field with the device towards the end of surgery [16,17]. The barcode scanning system is another technology, where a barcode is attached to every surgical sponge, so that it is verified and scanned at the end of surgery. A handheld barcode reader is used to scan each item before and after the surgery. This system is being practised across many hospitals in the United States [18]. A third technical innovation is utilising a magnetic metal tag on the surgical sponge that can be detected by a handheld detector [19].

Though these technical innovations serve their purpose, they levy a heavy expense onto the patient’s hospital bill. This mandates a cost-effective, cutting-edge novelty to avoid its incidence.

We have implemented a novel, yet simple, technique of handling the surgical sponge in this study. The pack of five sponges (tied at their tail end) is too voluminous to be overlooked while closing the abdomen. This technique is very affordable, efficient and time-limiting. Feedback from the surgical crew members was overwhelmingly positive. It is very easy to inculcate and execute in day-to-day surgical practice. Survey results have shown statistically significant improvements in the ease of sponge handling. There was negligible doubt on a retained surgical sponge from the surgical team during this study.

This new technique is not only easy to use but also comes as a handy, cost-cutting technique, apart from offering the surgical team a lower chance of error. 

Limitations of the study

Being a prospective observational study, there is no comparison of the incidence of gossypiboma cases with a control group. Lack of a control group makes it difficult to generalise the results of the study.

## Conclusions

Given the current scenario, where medicolegal scrutiny and social media exposure of the medical fraternity are looming large, there is a pressing need for health care providers to be on a zero-tolerance approach towards medical negligence. As with gossypiboma, which is a preventable error, measures to avoid its occurrence are to be instituted at every point during surgery. Sponge count, a responsibility, is not only vested upon the scrub nurse but also onto the entire surgical team.

The usage of novel techniques, such as the one utilized in this study, can be practised hassle-free even across small and medium hospitals. Such an approach not only minimises the incidence of gossypiboma but also provides a safer surgical environment, thereby giving the surgical team as well as the patient a peaceful postoperative period. This study has fulfilled its objective in providing a feasible alternative for sponge-handling during surgery.

## Figures and Tables

**Figure 4:**
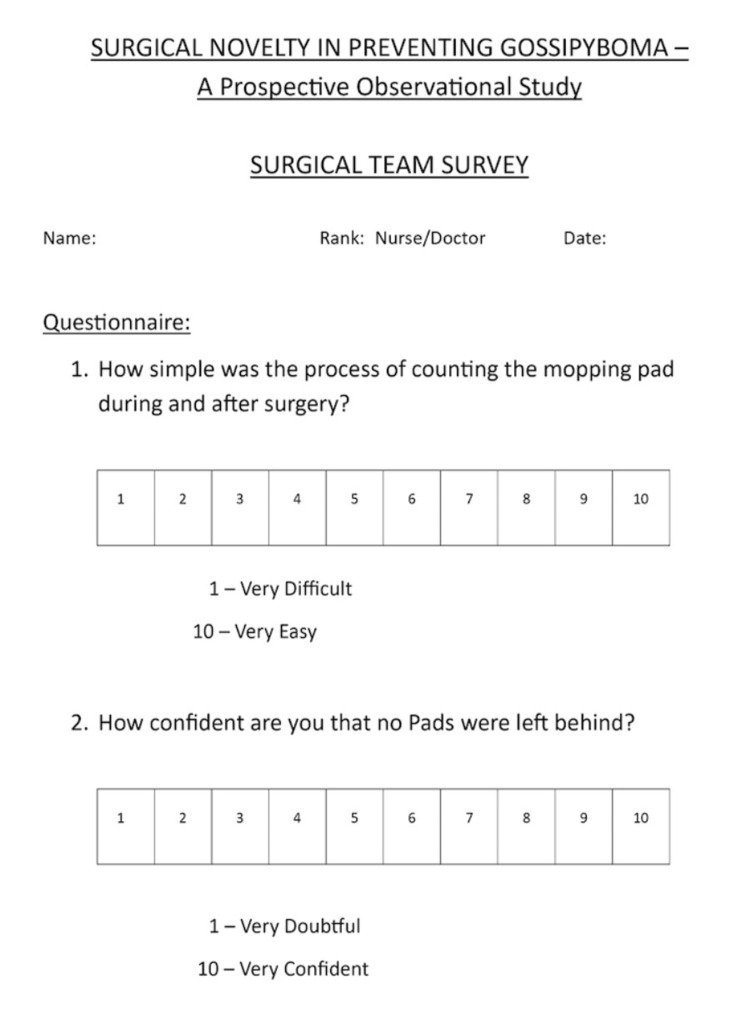
Survey Questionnaire proforma
